# Effects of photoperiod change on serum hormone level, hair follicle growth and antioxidant status in skin tissue of cashmere goats

**DOI:** 10.3389/fvets.2025.1548681

**Published:** 2025-03-19

**Authors:** Chenyu Mao, Xuelei Yin, Chu Wang, Xinran Huang, Jiawen Li

**Affiliations:** Faculty of Biological Science and Technology, Baotou Teacher's College, Baotou, China

**Keywords:** cashmere goat, photoperiod changed, hair follicle growth, hormone level, antioxidation state of skin

## Abstract

The growth of cashmere in goats was primarily influenced by natural photoperiod. However, whether artificially altering the photoperiod modified the rhythm of cashmere growth still required verification. In this study, the effects of photoperiod change on hormone secretion, hair follicle development, gene expression and skin antioxidant status of goats were studied in non-growth period of cashmere. Eighteen goats were randomly divided into three groups: control group (CG, natural photoperiod), short-day photoperiod group (SDPP, light 8 h/d, dark 16 h/d) and shortening photoperiod group (SPP, illumination duration gradually shortened from 16 h/d to 8 h/d). Experiment lasted for 60 days. Blood samples were taken weekly in first 30 days and every other day in last 30 days to determine hormone concentration. Skin samples were collected on 30 d and 60 d to determine hair follicle morphology, gene expression and skin antioxidant index. The results showed that SDPP and SPP increased the melatonin concentration on 34 d (*p* < 0.05) and 44 d (*p* < 0.05), and the epidermal growth factor concentration on 46 d (*p* < 0.05) and 50 d (*p* < 0.05), and the T3 concentration on 48 d and 56 d (*p* < 0.05), but decreased the prolactin concentration on 44 d (*p* < 0.05) and 56 d (*p* < 0.05), respectively. Additionally, on the 60 d, SDPP and SPP increased the depth of secondary hair follicle and the width of primary hair bulb (*p* < 0.05) and SPP increased the width of secondary hair bulb (*p* < 0.05). Furthermore, on the 60 d, SDPP up-regulated the *β*-catenin expression; SPP up-regulated the *β*-catenin, BMP2 and PDGFA expression (*p* < 0.05). Besides, on the 30 d, SDPP increased the activity of catalase (CAT) (*p* < 0.05) and decreased the content of malonaldehyde (MDA) (*p* < 0.05). On the 60 d, SPP increased the activities of total superoxide dismutase, both SDPP and SPP increased the activities of CAT and glutathione peroxidase (GPx) (*p* < 0.05), and decreased content of MDA in skin (*p* < 0.05). In addition, at 60 d, both SDPP and SPP up-regulated the gene expression of SOD1, GPx4 and CAT (*p* < 0.05). It can be seen that shortened the photoperiod affected the hair follicle activity by altering the secretion of hormone and mediating the expression of key genes, made hair follicle morphological changes. Meanwhile, short photoperiod improved the antioxidant capacity, created favorable conditions for cashmere growth.

## 1 Introduction

The process of cashmere shedding was complicated, which was influenced by many factors including environment, nutrition and heredity. In recent years, the research on cashmere growth had focused on natural conditions. The research showed that the hair follicle growth of Liaoning cashmere goats had obvious periodism: the hair follice development entered catagen from January, during which hair follicle depth declined until May. Then hair follice development entered telogen from June to Augest. Finally, hair follice development entered anagen from September till January (1). With the deepening of research, the researchers found that the hormone levels in cashmere goats were quite different during the cashmere flourish period and the latent period. Hormones including melatonin (MLT), prolactin (PRL) and insulin-like growth factor (IGF-I), epidermal growth factor (EGF), played a unique role and were interrelated. It was interesting that the other hormones secretion changed had a strong correlation with the MLT variation, which existed in many furred animals including cashmere goats (2). Study indicated that the goats implanted MLT regulated the secretion of PRL, and together affected the cashmere yield (3). Moreover, MLT injection played a role of trigger to increase IGF-1 concentration in Liaoning cashmere goats (4). Similar phenomena were obtained in Angora rabbits, the results showed the MLT synthase blocker also influenced other hormones secretion, and finally affected the molting cycle (5).

With the change of natural photoperiod, the signal pathways related to villus growth are activated. At present, Wnt/*β*-catenin, Notch and MAPK pathway were the main ones (6). *β*-catenin played a decisive role in Wnt/β-catenin pathway, which function was reflected in the formation and maintenance of villus matrix (7). In addition, *β*-catenin was also involved in the whole process of hair follicle development and cashmere periodic growth (8), which made *β*-catenin a key gene to study hair follicle morphology. Surprisingly, *β*-catenin and MLT seem to be related closely. Studies had shown that the expression of *β*-catenin was up-regulated after MLT implantation, meanwhile, the expression of Bone morphogenetic protein (BMP), a key gene downstream of the pathway, was also affected (9, 10). BMP gene family played a key role in the development of hair follicle: BMP2 regulated the processes of cell division and self-renewal and the maintenance of stem cells; BMP4 was widely expressed in hair follicle stromal cells and epidermis during embryonic hair follicle formation and postnatal hair follicle periodic growth (11). In addition, platelet-derived growth factor A (PDGFA) was also up-regulated after MLT injection (12). PDGF is a potent mitogen produced in a variety of cell types and is important for cell growth, proliferation and differentiation (13), among them, PDGFA is considered to contribute to hair follicle regeneration during hair cycle (14). This showed that the increase of MLT concentration activated the signal transduction and stimulated the cashmere growth. MLT could not only be used as a hormone to regulate the cashmere growth and activate the signal transduction, but also as an active molecule to change the state of hair follicles. Current research showed that MLT regulated the proliferation and apoptosis of many cells, including hair follicle stem cells and lymphocytes (15, 16). In addition, the variation of MLT level could significantly affect the free radical scavenging ability of mammals, which may be mediated by receptor signaling and the activation mechanism of antioxidant and anti-inflammatory factors (17–19). MLT played effective in relieving oxidative stress. Studies showed that exogenous MLT improved the gene expression and activity of glutathione peroxidase (GPx) and total superoxide dismutase (TSOD) in liver of mice (20). After the pineal gland (PG) was excised, the activity of GPx in intestinal tissues of mice decreased (21). However, no study had been done on the relationship between artificial shortening of illumination duration and skin antioxidant status, which was focused in present study.

Up till now, most studies adopt the method of injection exogenous MLT, there were few studies on the influence of artificial control of photoperiod on MLT secretion and hair follicle morphology. Besides, it is not known whether shortening photoperiod can increase the antioxidant capacity of skin, so as to make cashmere grow healthily and stably. Therefore, in present research, the effects of different photoperiods on hormone secretion level, signal pathway transduction and skin antioxidant status of cashmere goats were studied. It had both practical significance and economic benefits on cashmere goat breeding, and provided new ideas for improving cashmere yield and quality in case of the seasonal follicle activity can be changed by artificially controlling photoperiod.

## 2 Materials and methods

### 2.1 Ethics statement

Goats were obtained from the Experimental Farm of Inner Mongolia Agricultural University. All animal experiments were performed in accordance with Animal Ethics and Welfare Committee of Baotou Teachers’ College (AEWC-BTTC2023003).

### 2.2 Animal and experiment design

The experiment was carried out in the experimental farm of Inner Mongolia Agricultural University from April 18th to June 16th, 2023, lasted for 60 days, during which the natural photoperiod was gradually extended from light 9 h/ day to light 16 h/ day. A total of 18 infancy female Arbas cashmere goats (6 months to 8 months old) with similar body weight (9.58 ± 0.49 kg) were randomly divided into three groups, with 6 head per group and housed in 3 environmentally controlled rooms, respectively. Goats in the control group (CG) received the natural photoperiod; those in the short-day photoperiod group (SDPP) received 8 h light and 16 h dark per day, with light provided by natural light from 10: 00 to 18:00; and those in the shortening-day photoperiod group (SPP) received 16 h light and 8 h dark initially, then the illumination duration shortened gradually by 1 h per week to 8 h light and 16 h dark per day, with light provided by fluorescent lamps when sunlight vanished. All 3 groups were fed the same diets at 10:00 and 17:00 which were formulated to meet or exceed the nutrient requirements recommended by the National Research Council (NRC, 2007). Water was provided ad libitum.

### 2.3 Sample collection and preparation

Blood samples were collected at 4:00 weekly during the first 30 days, and every other day during the last 30 days for the analysis of hormone concentration. The serums were harvested after centrifugation for 20 min at 3000 × g and then frozen at −20°C. Skin hair follicle samples were collected on the 30 d and 60 d of the experiment. Hemostatic forceps was used to pick up the skin tissue near the scapula of goats, and fat was avoided as much as possible. Then, a total of 1.5 cm2 skin samples were taken by surgical scissors and divided into two parts, one part was immediately put into formalin for morphological observation, and the other part was stored in liquid nitrogen for determination of antioxidant index and mRNA extraction.

### 2.4 Assay of hormone in serum

Hormone concentration, including MLT, PRL, IGF-1, EGF, thyroxine 3 (T3), and thyroxine 4 (T4), were determined with commercial ELISA kits (Ruixin Biological Technology Co., Ltd. Quanzhou, China) according to the manufacturer’s instructions.

### 2.5 Determination of antioxidant indexes in tissue samples

After the depilated, the skin samples were minced and homogenized with ice-cold saline (wt/vol, 1:9), then centrifuged at 4000×g for 15 min at 4°C. Total antioxidant capacity (TAOC), MDA content and the activity of enzymes, including tTSOD, GPx, and catalase (CAT) were measured by spectrophotometric method according to the instructions of the commercial kits (Nanjing Jiancheng Institute of Bioengineering, Nanjing, China). The activity of TSOD, GPx, and CAT was expressed as activity unit per milligram of tissue protein (U/mg protein). The concentration of MDA was expressed as nanomole per milligram of tissue protein (nmol/mg protein). TAOC capacity was expressed as micromole (μmol) Trolox equivalent per gram protein of homogenates (μmol/g protein).

### 2.6 Morphological observation of hair follicle

Square skin tissue was preserved in formalin. Following a 24-h rinse under running water, ethanol dehydration was performed. Then, the samples were made transparent using xylene, followed by paraffin infiltration, and finally embedded in hard paraffin. After that, the specimens were then sectioned using a paraffin microtome, adjusting the section thickness to 5–7 μm, with continuous sections taken at different sites. The usable specimens were stained, involving the steps of deparaffinization, staining, and dehydration followed by mounting. The observations included measurements in 10 different fields of view under an electron microscope for the following parameters: primary follicle depth (μm) (vertical depth, the same as secondary follicle depth), primary follicle bulb width (μm), secondary follicle depth (μm), and secondary follicle bulb width (μm).

### 2.7 Total mRNA extraction and quality determination

Total RNA was obtained using Trizol Reagent according to the manufacturer’s protocol. The extracted mRNA was quantified spectrophotometrically and the OD260/OD280 was used for evaluation of quality. Subsequently, the total mRNA was treated with DNase I (TaKaRa Biotechnology Co. Ltd., Dalian, China) to remove gDNA, and then reverse-transcribed into cDNA on LifeECO (Bori Technology Co., Ltd. Hangzhou, China) using a Prime Script RT™ Master Mix kit (TaKaRa). The reactions were performed with incubation for 15 min at 37°C, followed by 5 s at 85°C.

### 2.8 Quantitative RT-PCR analysis

The resulting cDNAs were used in quantitative RT-PCR (qRT-PCR) reactions. The qRT-PCR for target genes and housekeeping genes (*β*-actin, B2M and YWHAZ) was performed in triplicate using the LightCycler® 96 Real-Time PCR Design & Analysis System (ROCHE Ltd., Basel, Switzerland) with a SYBR® Premix Ex Taq™ Kit (TaKaRa). The qRT-PCR was performed using 20 μL reactions that contained 10 μL of 2 × TB Green Premix Taq II, 0.8 μL of each primer (10 μM), 6.4 μL of water and a 2 μL cDNA template. This was performed with the following cycling conditions: 95°C for 30 s (hold stage), followed by 40 cycles of 95°C for 15 s and 60°C for 30 s (PCR stage), then 95°C for 10 s, 60°C for 1 min, and 95°C for 15 s.

The specific sequences of primers are listed in Table 1. *β*-2-microglobulin (B2M), tyrosine 3-monooxygenase (YWHAZ) and beta-actin (*β*-actin) were used to normalize the expression data according to the method reported by Wang (22). The relative quantity of target gene mRNA was expressed as 2^-∆∆ct^ using the relative comparative threshold cycle method as described previously (23), and for the normalization of the RT-qPCR data, the geometric mean Ct of 3 reference genes was used (24).

**Table 1:** Primer sequences.

Gene name		Primer sequence	Gene no.	Size
*β*-catenin	F	GCTGATTTGATGGAGCTGGA	XM_018066894.1	100
	R	TCATACAGGACTTGGGTGGT		
BMP2	F	AAGAGGCATGTGCGGATTAG	NM_001287564.1	179
	R	TTGCCGCTTTTCTCTTCTGT		
BMP4	F	GCTCTACGTGGACTTCAGTG	NM_001285646.1	124
	R	TGGTTGGTTGAGTTGAGGTG		
FGF5	F	GGCACTTGCATGGAGTTTTCC	NM_001291973.1	112
	R	AGTGGGCATCGGTTTCCATC		
PDGFA	F	CAGTCAGATCCACAGCATCC	XM_018040679.1	116
	R	CAGACTGGTTTCCAAAGGCT		
SOD1	F	ATCCACTTCGAGGCAAAGGG	NM_001285550.1	104
	R	GCACTGGTACAGCCTTGTGTA		
SOD2	F	TCAATAAGGAGCAGGGACGC	XM_005684984.1	85
	R	AGCAGGGGGATAAGACCTGT		
GPx1	F	ACATTGAAACCCTGCTGTCC	XM_005695962.2	216
	R	TCATGAGGAGCTGTGGTCTG		
GPx4	F	TTCCCTTGCAACCAGTTTGG	NC_030814.1	105
	R	TCATCCATTTCCACAGAGGGT		
CAT	F	CACTCAGGTYCGGGATTTCT	GQ_204786.1	159
	R	ATYCGGGAGCCATATTCAGG		
*β*-actin	F	ACTGGGACGACATGGAGAAGA	U39357	199
	R	YCGTACAGGGACAGCACAG		
B2M	F	GGTGCTGCTTAGAGGTCTCG	NM_001009284	109
	R	ACGCTGAGTTCACTCCCAAC		
YWHAZ	F	TGTAGGAGCCCGTAGGTCATCT	AY970970	102
	R	TTCTCTCTGTATTCTCGAGCCATCT		

### 2.9 Statistical analysis

Data were analyzed by repeated measures ANOVA using the generalize linear model (GLM) procedure of SPSS for Windows (Version 27.0). Differences among the treatment means were detected using Duncan’s test, and considered significant at *p* < 0.05. Data are presented as mean ± SD.

## 3 Results

### 3.1 Effect of photoperiod change on hormone secretion of cashmere goats

**Figure 1:**
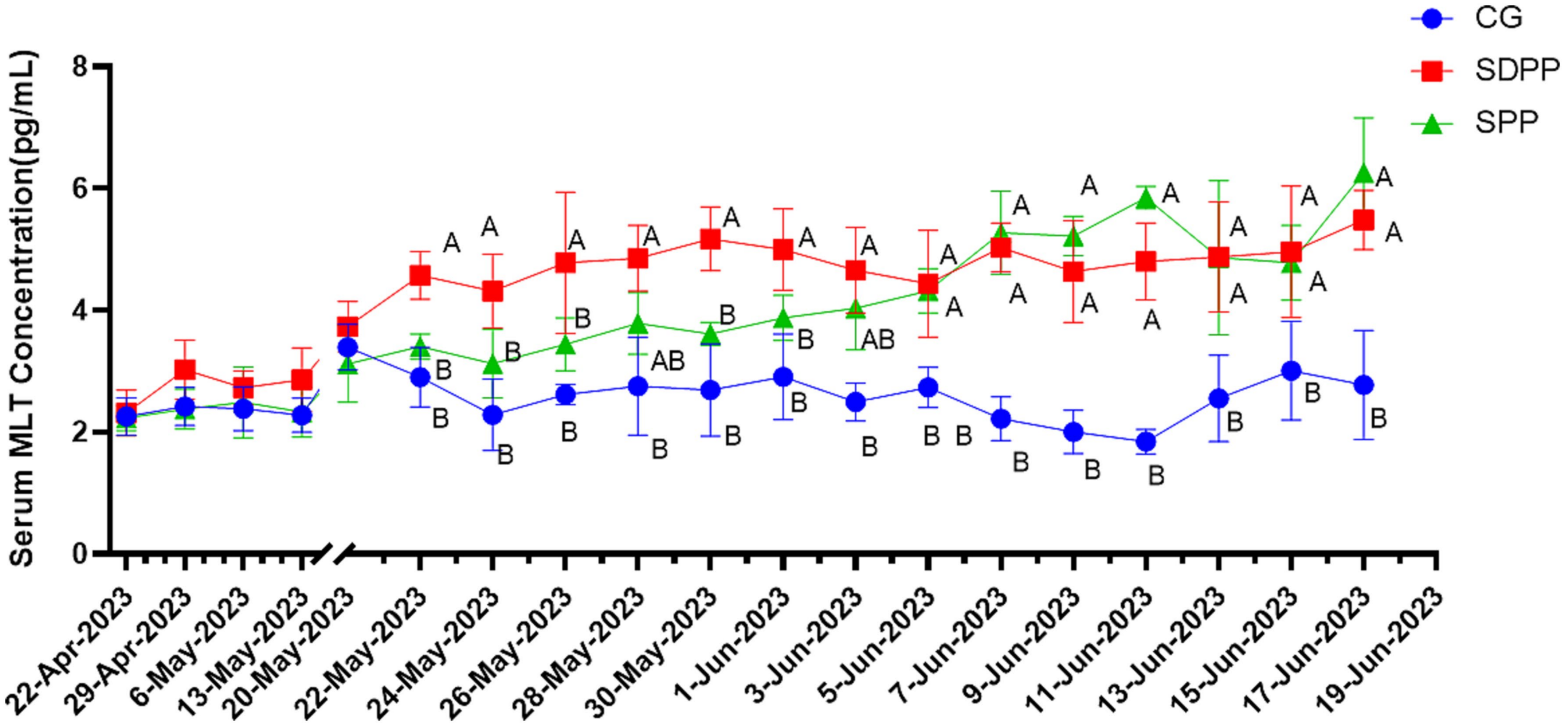
Effect of photoperiod change on MLT secretion. Different letters indicate significant difference at *p* < 0.05 among the groups; CG, control group; SDPP, short-day photoperiod; SPP, shortening-day photoperiod.

As shown in Figure 1, goats in SDPP group had a higher MLT concentration on 34 d of the experiment (*p* < 0.05). Then the MLT concentration maintained a high level until the end (*p* < 0.05). On the 44 d, the serum MLT concentration of goats in SPP group began to increase (*p* < 0.05) and continued to the end of the experiment (*p* < 0.05).

**Figure 2:**
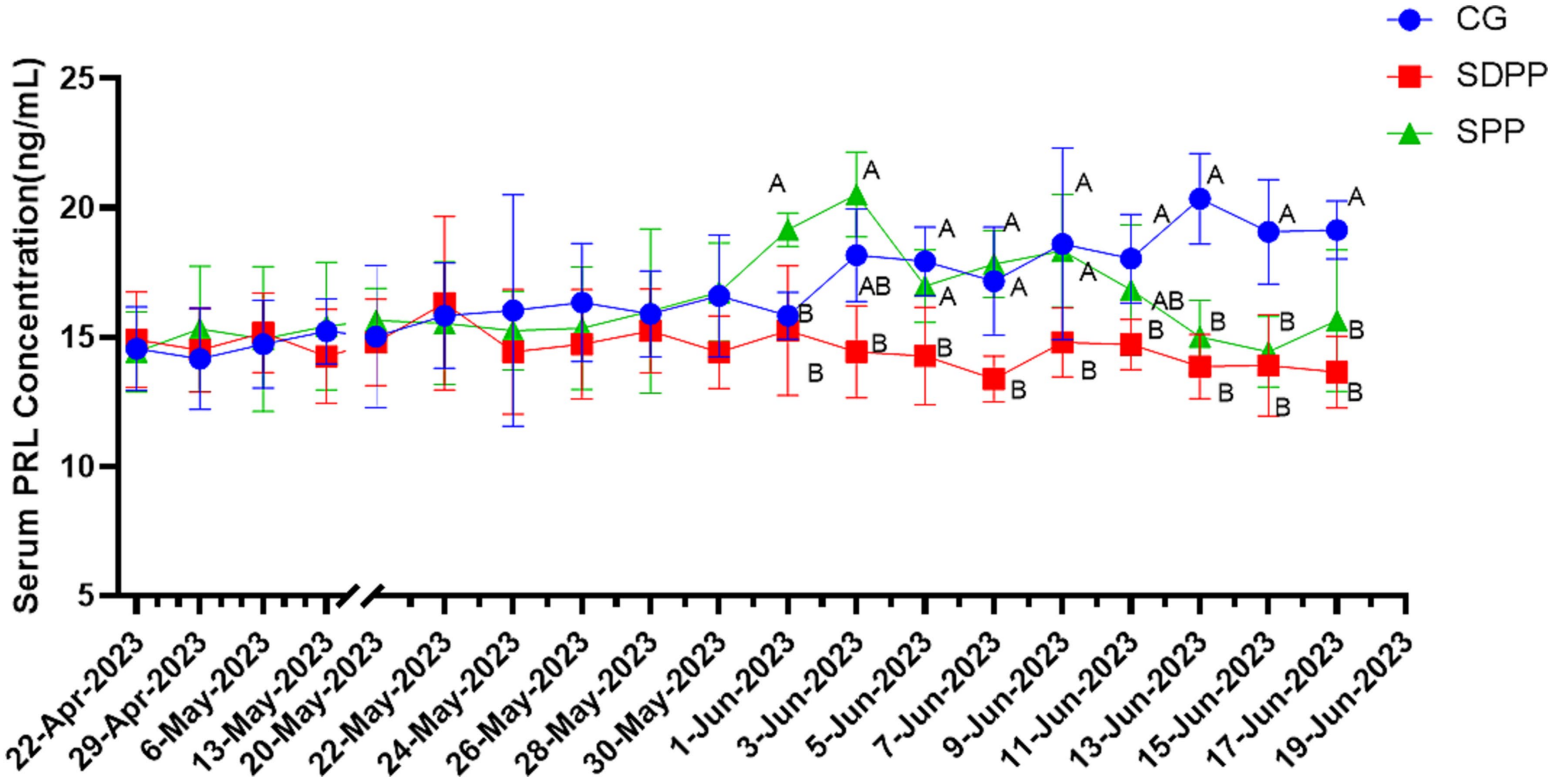
Effect of photoperiod change on PRL secretion. Different letters indicate significant difference at *p* < 0.05 among the groups; CG, control group; SDPP, short-day photoperiod; SPP, shortening-day photoperiod.

As shown in Figure 2, the serum PRL concentration of goats in CG gradually increased, while the serum PRL concentration of cashmere goats in SDPP group remained at a low level, and it was lower than that in the CG on the 44 d of the experimental (*p* < 0.05). The serum PRL concentration of SPP goats increased in the middle of the experiment and reached the peak on the 46 d (*p* < 0.05). After that, the concentration began to decrease. On the 56 d, the concentration of serum PRL was lower than that of the CG (*p* < 0.05), and maintained to the end of the experiment.

**Figure 3:**
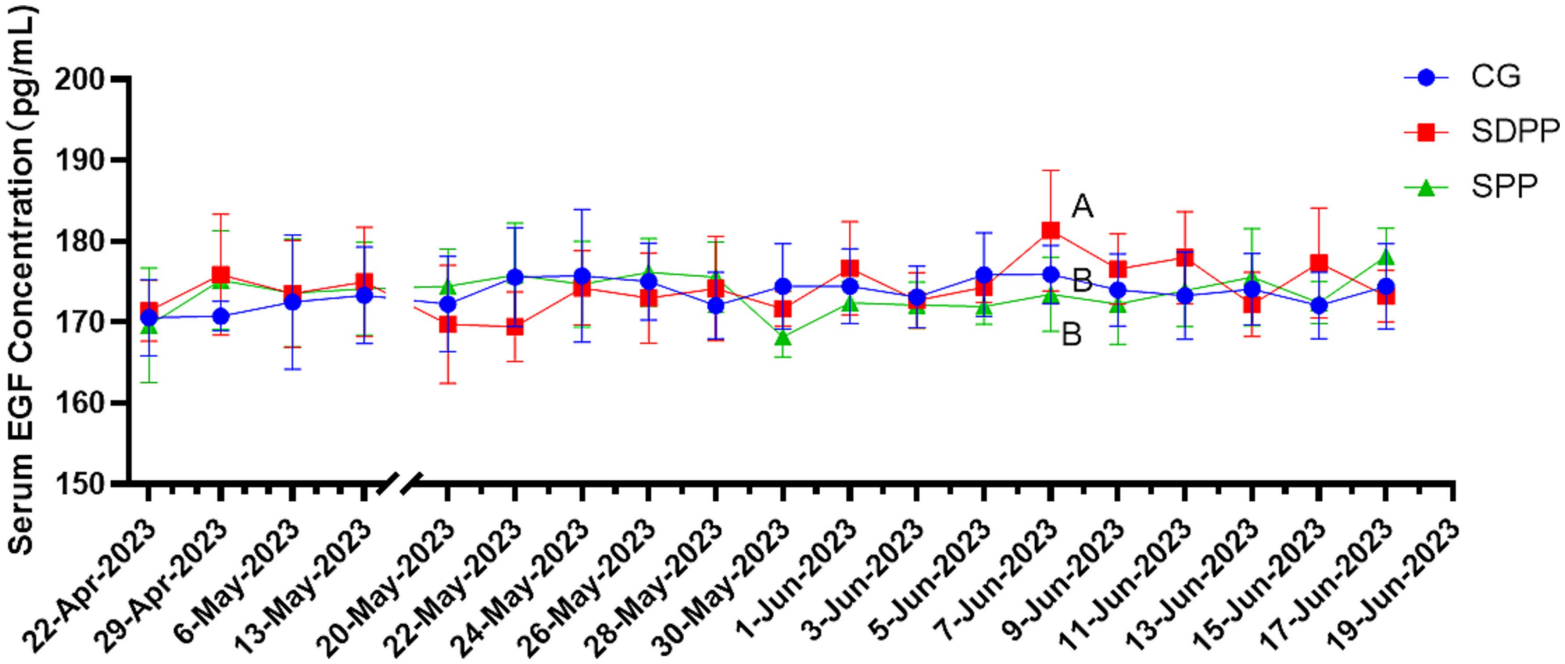
Effect of photoperiod change on IGF-1 secretion. Different letters indicate significant difference at *p* < 0.05 among the groups; CG, control group; SDPP, short-day photoperiod; SPP, shortening-day photoperiod.

Figure 3 showed the effect of photoperiod changes on goat IGF-1 secretion. Although the serum IGF-1 concentration in SDPP group increased on 42 d and 48 d of the experiment (*p* < 0.05), over the entire experimental period, photoperiod changed had no significant effect on IGF-1 secretion in goats. There was no difference in serum IGF-1 concentrations between the photoperiod treatment groups and the CG (*p* > 0.05).

**Figure 4:**
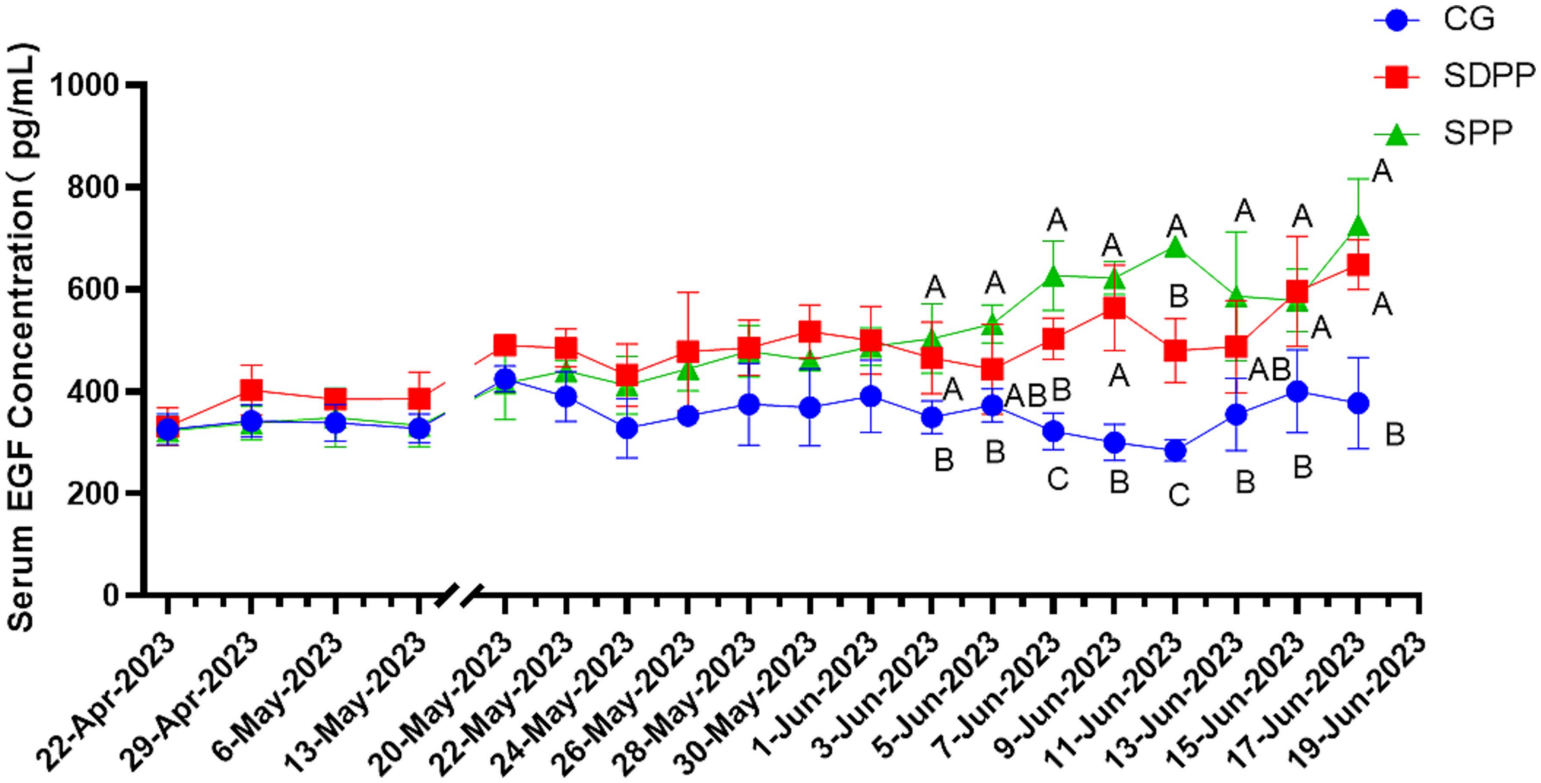
Effect of photoperiod change on EGF secretion. Different letters indicate significant difference at *p* < 0.05 among the groups; CG, control group; SDPP, short-day photoperiod; SPP, shortening-day photoperiod.

Figure 4 listed the changes of EGF concentration in three groups. On the 46 d, the serum EGF concentration in the SDPP group increased (*p* < 0.05), and kept high secretion until the end. The goats in SPP group kept high concentration of EGF from 50 d (*p* < 0.05) until the end.

**Figure 5:**
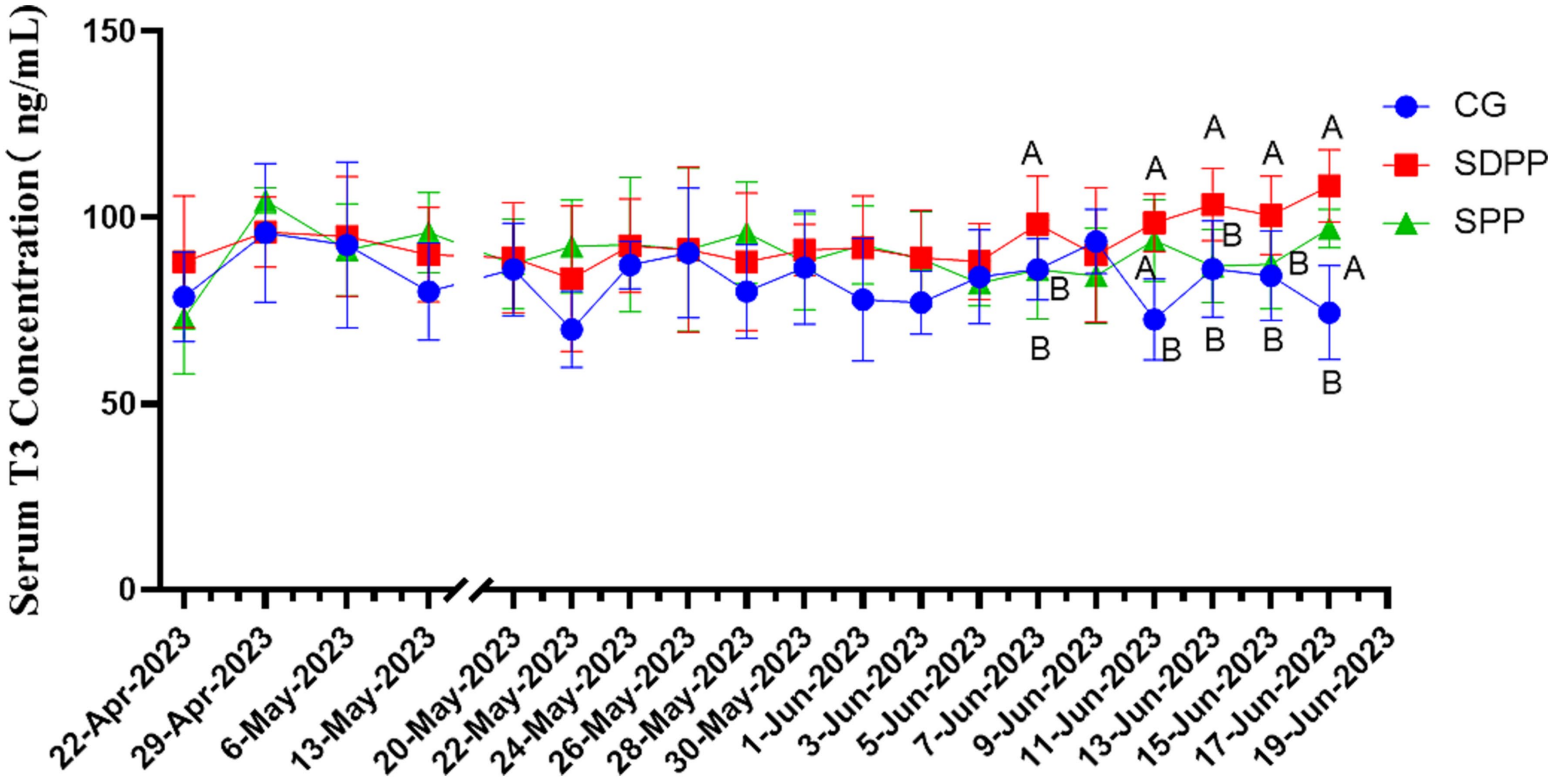
Effect of photoperiod change on T3 secretion. Different letters indicate significant difference at *p* < 0.05 among the groups; CG, control group; SDPP, short-day photoperiod; SPP, shortening-day photoperiod.

As shown in Figure 5, on 48 d, T3 concentration in SDPP increased (*p* < 0.05), but the secretion fluctuated in the following week. It was not until the 56 d that the serum T3 concentration in this group was higher than that of the CG (*p* < 0.05), and remained in a high secretion state until the end. T3 concentration in the SPP group increased on the 60 d (*p* < 0.05).

**Figure 6:**
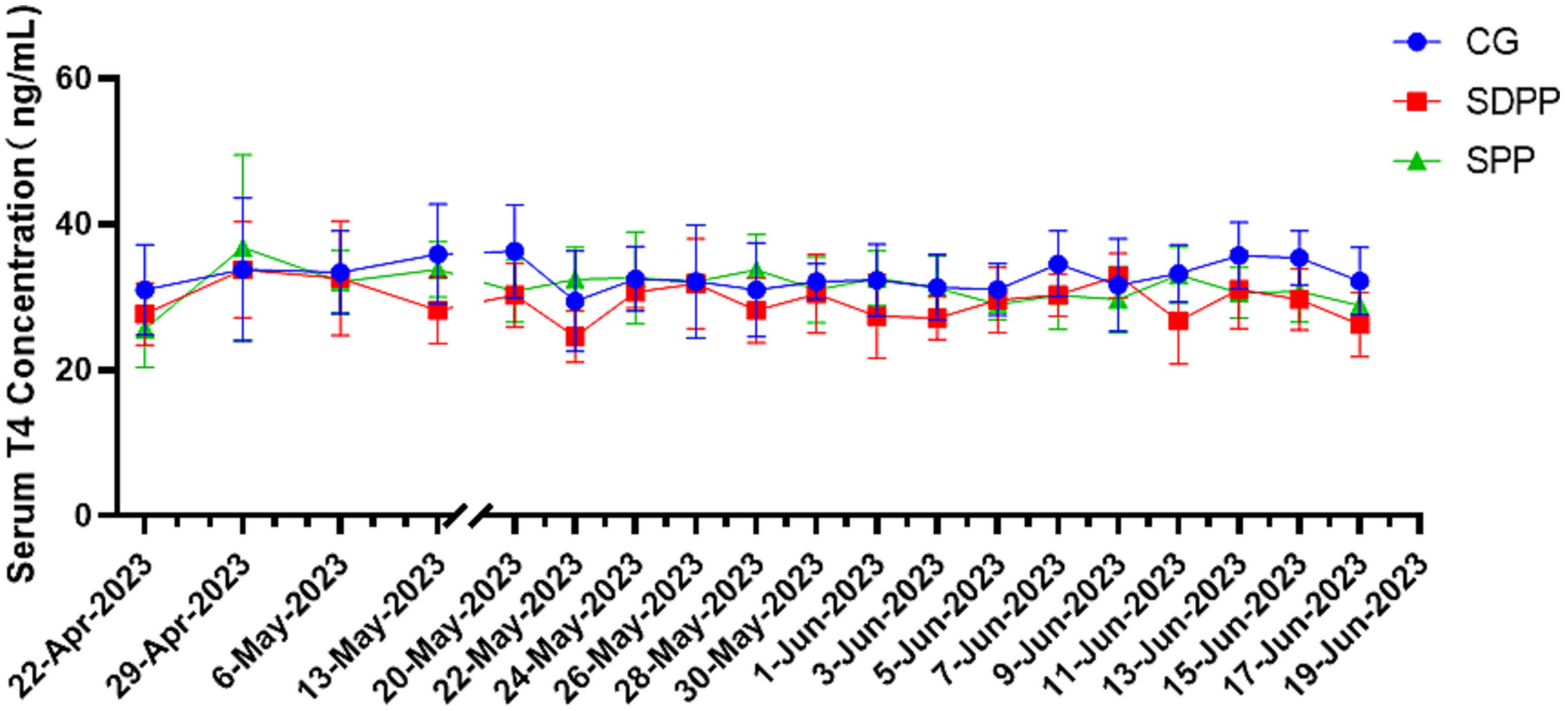
Effect of photoperiod change on T4 secretion. Different letters indicate significant difference at *p* < 0.05 among the groups; CG, control group; SDPP, short-day photoperiod; SPP, shortening-day photoperiod.

Figure 6 showed the effect of photoperiod change on serum T4 secretion of cashmere goats. The results indicated that change of photoperiod did not affect the secretion of T4 (*p* > 0.05).

### 3.2 Effect of photoperiod change on hair follicle morphology of cashmere goats

**Figure 7:**
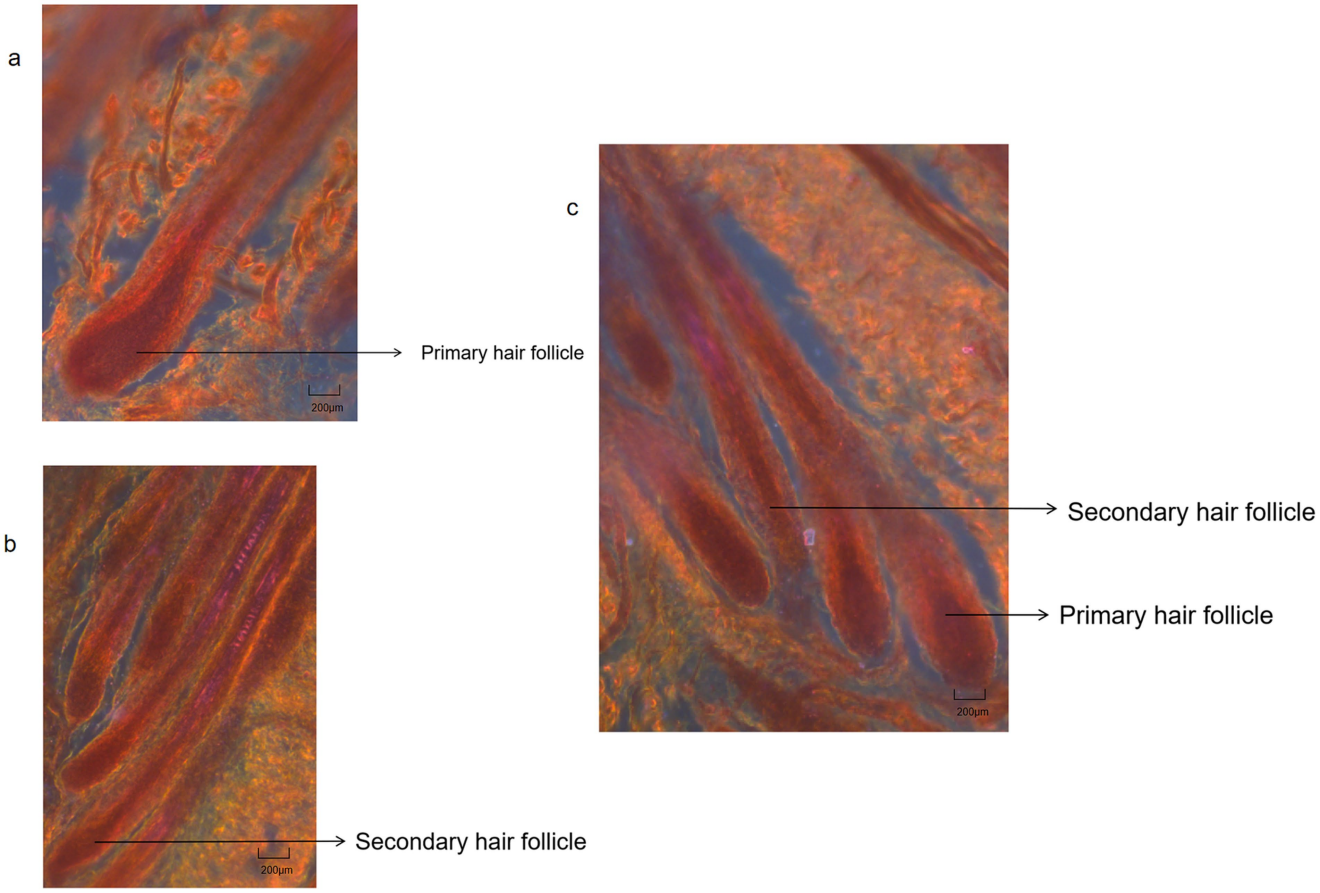
Hair follicles histological structure of cashmere goat. **(A)** Primary hair follicles structure of cashmere goats (4X); **(B)** Secondary hair follicles structure of cashmere goats (4X); **(C)** Primary hair follicles and secondary hair follicles structure of cashmere goats (4X).

**Figure 8:**
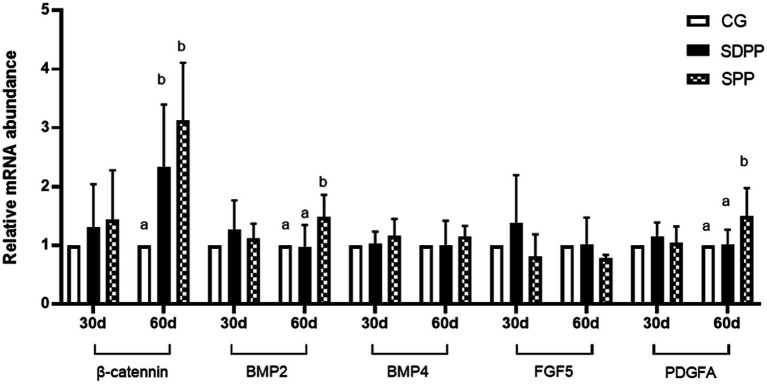
Effect of photoperiod change on relative gene expression of hair follicle development related genes. Bars carrying different letters (a, b) were significantly different (*p* < 0.05) (mean standard error, *n* = 6); CG, control group; SDPP: short-day photoperiod; SPP, shortening-day photoperiod.

**Figure 9:**
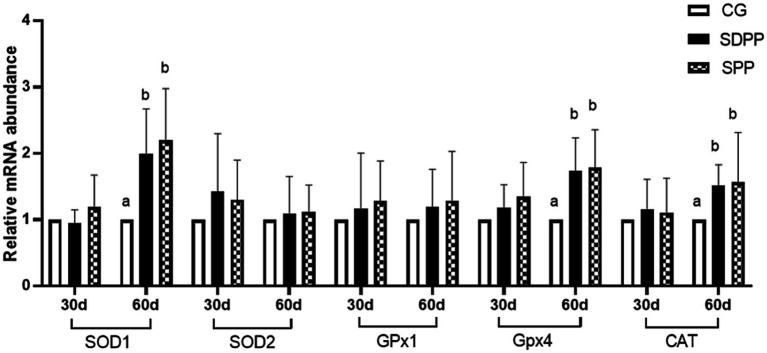
Effects of photo period change on expression of antioxidant relative genes in skin tissue. Bars carrying different letters (a, b) were significantly different (*p* < 0.05) (mean standard error, *n* = 6); CG, control group; SDPP, short-day photoperiod; SPP, shortening-day photoperiod.

Primary hair follicles and secondary hair follicles of cashmere goat skin tissue were shown in Figures 7–9. The effects of photoperiod changes on the morphology of cashmere goat follicles were shown in Table 2. At the 30 d of the experiment, there were no significant differences in all indexes among the three experimental groups (*p* > 0.05). At day of 60, compared with the CG, the depth of secondary follicles in SDPP and SPP group increased significantly (*p* < 0.05). For follicle bulb width, the SPP treatment increased both primary and secondary follicle bulb widths (*p* < 0.05), while SDPP increased secondary hairball width (*p* < 0.05).

**Table 2:** Effects of photoperiod change on hair follicle morphology (μm).

Items	Groups			*p*-value
	CG	SDPP	SPP	
Day 30				
Primary hair follicle depth	953.9 ± 28.4	1010.1 ± 65.5	988.8 ± 42.7	0.951
Secondary hair follicle depth	505.7 ± 46.32	514.2 ± 44.4	507.0 ± 16.2	0.918
Primary hairball width	155.5 ± 3.20	155.5 ± 5.10	154.1 ± 3.49	0.467
Secondary hairball width	46.8 ± 3.02	44.5 ± 2.45	45.8 ± 4.66	0.553
Day 60				
Primary hair follicle depth	994.9 ± 86.5	1001.1 ± 42.9	1052.3 ± 76.1	0.321
Secondary hair follicle depth	523.5 ± 63.4^b^	744.7 ± 28.9^a^	788.4 ± 18.7^a^	<0.01
Primary hairball width	144.9 ± 2.66^b^	148.4 ± 6.17^b^	167.6 ± 6.51^a^	<0.01
Secondary hairball width	44.19 ± 4.48^b^	60.55 ± 4.37^a^	58.12 ± 2.52^a^	0.042

Different letter showed that the difference was significant in the same column (*p* < 0.05).

### 3.3 Effect of photoperiod change on gene expression related to hair follicle development

Figure 8 showed the effect of photoperiod change on the expression of genes related to hair follicle development in cashmere goats. On the 30 d, photoperiod variation did not affect the expression of genes (*p* > 0.05). At the 60 d of the experiment, compared with the CG, the SDPP group up-regulated the gene expression of *β*-catenin (*p* < 0.05). For SPP group, the change of photoperiod up-regulated the gene expression of *β*-catenin, BMP2 and PDGFA in hair follicles (*p* < 0.05).

### 3.4 Effect of photoperiod change on antioxidant indicators

The effect of photoperiod regulation on the antioxidant status of skin tissue was shown in Table 3. At 30 d, the SDPP treatment enhanced the activity of CAT in skin tissue (*p* < 0.05) and decreased the content of MDA (*p* < 0.05), but the SPP treatment had no effect (*p* > 0.05). At 60 d, compared with the CG, the activities of CAT and GPx in goat skin tissue under SDPP increased (*p* < 0.05), and the content of MDA decreased (*p* < 0.05). The activities of T-SOD and GPx enzymes were increased (*p* < 0.05) and the content of MDA was decreased (*p* < 0.05) in the SPP group.

**Table 3:** Effects of photoperiod change on skin antioxidant indexes.

Items	Groups			*p*-value
	CG	SDPP	SPP	
Day 30				
T-SOD/(U/mg prot)	55.19 ± 8.54	55.42 ± 3.32	51.30 ± 6.76	0.693
CAT/(U/mg prot)	23.18 ± 2.30^b^	27.58 ± 3.77^a^	21.51 ± 3.63^b^	0.041
MDA/(nmol/mg prot)	10.99 ± 1.68^a^	7.13 ± 1.34^b^	10.75 ± 2.35^a^	0.042
GPx/(U/mg prot)	100.9 ± 9.4	95.4 ± 13.62	105.3 ± 16.1	0.586
TAOC/(U/mg prot)	1.67 ± 0.31	1.94 ± 0.33	1.78 ± 0.26	0.49
Day 60				
T-SOD/(U/mg prot)	60.31 ± 5.48^b^	58.08 ± 7.10^b^	77.10 ± 5.19^a^	0.797
CAT/(U/mg prot)	22.06 ± 1.04^b^	27.74 ± 2.91^a^	28.51 ± 2.08^a^	0.026
MDA/(nmol/mg prot)	11.41 ± 2.26^a^	6.31 ± 0.73^b^	6.65 ± 1.27^b^	0.012
GPx/(U/mg prot)	95.9 ± 9.0^b^	114.7 ± 8.7^a^	111.3 ± 8.0^a^	0.049
TAOC/(U/mg prot)	1.53 ± 0.23	1.94 ± 0.34	1.81 ± 0.20	0.125

Different letter showed that the difference was significant in the same column (*p* < 0.05).

### 3.5 Effect of photoperiod change on the relative expression of mRNA related to antioxidant status

Figure 9 showed the effect of photoperiod changed on the expression of antioxidant genes in cashmere goat skin. At 30 d, photoperiodic variation did not affect the gene expression (*p* > 0.05). On the 60 d of the experiment, compared with the CG, both SDPP and SPP treatment up-regulated the gene expression of SOD1, GPx4 and CAT in skin tissue (*p* < 0.05).

## 4 Discussion

### 4.1 Photoperiod variation affect hormone secretion

As seasons change, photoperiod variation is converted into electrical signals on the retina, which is then transmitted to the PG. This results in the release of norepinephrine from postganglionic sympathetic nerve endings into the PG, thereby maintaining the MLT status via β1-adrenergic receptors. Numerous studies had shown that photoperiods directly influenced the secretion of MLT, displaying significant diurnal rhythm changes, with the concentrations of MLT peaking between 2:00 and 3:00 AM (25). Additionally, the concentration of MLT fluctuated throughout the year; compared to summer, the secretion of MLT increased during winter nights and lasted longer. Thus, the prolonged high levels of MLT at night represented the length of the night, which could signal seasonal changes and regulate seasonal activities (26). Research had confirmed that MLT had the ability to enhance the growth of fur. As early as the 1980s, researchers improved the fur density and yield in minks through the injection of exogenous MLT (27). In studies on rabbit, Lanszki (5) found that injecting exogenous MLT increased the number of hair follicles and length of the fibers. In research on cashmere goats, Liu (28) used light-blocking technique and subcutaneous MLT injections to change hair follicle development from only in autumn to throughout the whole year, increasing cashmere yield by 70%. Similar phenomena were observed in Liaoning white cashmere goats: after the winter solstice, goats implanted MLT showed a significantly higher yield than the control group, which received natural photoperiod. This was supported by exogenous MLT, experienced a slower cessation of cashmere growth (29). Seasonal cashmere production phenomena also appeared in Spanish goats, where studies indicated that, compared to natural photoperiod, implanted MLT in cashmere goats could prolong the cashmere growth cycle, increasing spring cashmere yield (30). Most of these studies were based on implanted MLT, and experiments solely altering the photoperiod were rare. In present experiment, shortening the photoperiod resulted in increased serum MLT concentrations. Sustained, high concentrations of MLT could alter the original signaling pathways, eliciting corresponding responses in the organism.

PRL is primarily secreted by the anterior pituitary gland and promotes mammary gland development in mammals. However, for hair follicle cells, high concentrations of PRL inhibit secondary hair follicles activities (31). The secretion of PRL was significantly affected by natural photoperiod, with a correlation between blood PRL content and the duration of light exposure, showing noticeable concentration variations in spring and autumn (32). The present study validated the theory. In the CG, goat blood PRL concentrations increased, possibly due to the experiment being conducted at the junction of spring and summer, when natural light duration gradually lengthens. In the SDPP, blood PRL concentrations decreased in the later stages of the experiment. Similarly, a comparable phenomenon occurred in the SPP group, although the hormone concentration increased in the middle, it was significantly lower than that in CG group in the end, which could be related to the increase in MLT concentration caused by short light exposure. Numerous experiments had shown that MLT can affect the secretion of PRL in animals. In a study of sheep, researchers demonstrated that serum MLT concentrations were inversely correlated with PRL secretion, i.e., high MLT concentrations could inhibit PRL secretion, similarly, exogenous MLT injections could reduce PRL concentrations from May to July (33). Foldes (34) found that long-term MLT injections in sheep led to reduced blood PRL concentrations. Furthermore, investigator found that implanting MLT inhibited peak daytime blood PRL concentrations and reduced overall daily concentrations (35).

The PRL concentration in the blood of goats in SPP group showed a phenomenon of rising first and then declining, which was probably due to cashmere goats had adapted to illumination variation. Therefore, in present study, the serum PRL concentration of cashmere goats in SPP group increased in the middle period and then began to decline, which was may also related to the increase in MLT concentration. Additionally, research on Shanbei cashmere goats indicated that the decreasing photoperiod treatment combine MLT injection significantly affected the PRL secretion (36). This was because the treatment affects the concentration of MLT, which in turn regulated the secretion level of PRL, which was consistent with the results of present study.

IGF-1 is mainly produced by the liver of animals and has the function of stimulating cell differentiation and proliferation and inhibiting protein degradation. The results of present sduty showed photoperiod variation did not alter the secretion of IGF-1, which was different from other studies. In study of native goats in Chaharmahal, researchers found that after the light-controlled conditions, the concentration of IGF-1 in the experimental group was significantly higher than that in the control group, regardless of the telogen or anagen phase of hair follicle (37). On the contrary, mice undergoing long-day photoperiod increased the concentration of IGF-1 in the serum and upregulate the expression of IGF-1 in skin hair follicles, but after treatment, both concentration and expression decreased significantly (32). Althouth whether photoperiod changes could affect the concentration of IGF-1 in blood was still controversial, it could be confirmed that IGF-1 played an important role in hair follicle development. The mechanism of this action might be through the cellular signaling pathway involving its receptor (IGF-1R) to stimulate the proliferation of hair follicle cells. Bai (38) found that the expression of IGF-I mRNA reached its highest level during the hair follicle growth phase, and in the meantime, the number of binding sites between IGF-IR and IGF-I in the hair papilla, dermal sheath, internal root sheath, and external root sheath of the cashmere goat hair follicle growth cells and keratinocytes was the highest.

EGF is an important signaling molecule in the process of hair follicle development. Studies had shown that EGF might have different effects on hair follicle cells at different stages of development. Silva (39) found that EGF inhibited the development of hair follicles in the early stage of growth, however, at other stages, EGF promoted the growth of hair follicle cells, accelerating the transition to the next developmental stage. Moreover, *in vitro* hair follicle cell culture, EGF effectively promoted its growth. The results of present study could be explained by this mechanism, that is, SDPP and SPP treatments increased the secretion level of MLT, and high concentrations of MLT could stimulate the secretion of EGF, made the concentration of EGF increased in the end of the experiment.

Thyroxine is a hormone secreted by the thyroid gland that has various physiological functions, including influencing tissue differentiation and metabolism in animals. Studies had shown that thyroxine was significantly affected by changes in photoperiod and had an impact on hair follicle development, though many research results were inconsistent. In the study on Australian sheep, Souza (40) found that during long photoperiods, the concentrations of T3 and T4 in the blood increased. In addition, Abecia (41) divided sheep into three groups, each exposed to different natural photoperiods. The results showed that in July, the T4 concentration was significantly lower than that in the others groups, and the yield of cashmere and follicle density increased. In present study, SDPP and SPP increased T3 concentration at the end. This indicated that the effect of MLT on T3 secretion levels could produce different results depending on the age and sex of the experimental subjects. In research on the regulation of cashmere growth patterns by MLT in Inner Mongolian cashmere goats, Duan (42) found that under natural conditions, there was a significant positive correlation between blood MLT and T4 concentrations, but T3 showed no changes either under natural conditions or with subcutaneous MLT implants. In present study, there were no differences in T4 throughout the entire period, which greatly differs from previous experimental results. It was difficult to explain the effect of artificially changing the photoperiod on thyroxine secretion in the body. One reason might be the difference between the concentration of thyroxine in the blood and that in the hair follicles. Another reason could be that the ratio of DIO2/DIO3 regulated by MLT produced different results depending on whether the hair follicle was in active period, as DIO2 and DIO3 were the most critical enzymes for the interconversion of T3 and T4 (43). Regardless of the reason, further experiments are needed to explore and prove these findings.

### 4.2 Photoperiod variation affect hair follicle morphology

Under natural conditions, the activity of hair follicles had a certain periodicity: primary follicles and secondary follicles revived before the shedding of cashmere fibers in April, the activity of primary follicles and secondary follicle peaked in summer and winter, respectively, and then decreases (32). In addition, study had indicated that the seasonal changes in the hair follicle development of cashmere goats may be due to the gradual shortening photoperiod under natural conditions rather than constant short photoperiod (30). Therefore, the SPP group was built in present study to ensure the scientificness of the research. In present experiment, on the 30 d, the change of photoperiod had no effect on the morphology of hair follicles, possibly due to the activity of the hair follicle in each group had not adapted to the change in illumination exposure yet. Due to differences in breed, sex, and age, the activity of secondary follicles varied in cashmere goats, but the general pattern was low activity in the spring and summer, a subsequent rise in the autumn, and peaked at winter when light exposure was the shortest, followed by decrease and followed a dormant period, and reactivation in the next spring. Additionally, Fans (44) experiments on the longitudinal sections of hair follicles from the same long-haired cashmere goats in different months showed that the depth of secondary follicles increased in autumn, supporting the conclusion that secondary follicle depth increased as light exposure decreases. Changes in hormones induced by altered light exposure could also explain the changes in follicle depth and hair bulb width: on one hand, high concentrations of PRL in the mid phase initiated the active status of secondary follicles, altering both follicle depth and hair bulb width. On the other hand, high concentrations of MLT also caused changes in follicle activity. Studies had shown that implanting MLT in the spring successfully induced cashmere re-growth in the autumn, because MLT increased the depth of secondary follicles and widened the hair bulbs mainly (7). It is noteworthy that in studies on cashmere goat, researchers had found that MLT was likely to promote cashmere growth by binding to the nuclear receptor RORa (45). In present experiment, the SDPP and SPP treatment promoted the secretion of MLT, thereby changing the activity of secondary hair follicles, increasing the width of the secondary hair bulbs. Data showed that the activity cycle of primary hair follicles in cashmere goats may not be annual (44), therefore, changes in photoperiod did not impact their activity. The results of present experiment confirmed this conclusion: there were no differences in the depth of primary follicles among the groups but it was certain that light exposure time was an important factor affecting the activity of hair follicles in cashmere goats.

### 4.3 Photoperiod variation affect gene expression

Changes in photoperiod altered expression of key genes in signaling pathways for cashmere growth. Current research indicated that MLT activated hair follicle development by regulating gene expression. Mu Qing’s research (46) showed that the implantation of exogenous MLT regulated the expression of Wnt10b and *β*-catenin, which were key genes in Wnt signaling pathway and regarded as activators of hair follicle regeneration, playing an important role in the transition of hair follicles from the catagen phase to the telogen phase. Natural changes in light exposure also affected the expression of Wnt10b and *β*-catenin. Wang (9) divided goats into two groups: control group under natural light and experimental group under constant short-day photoperiod. The results showed that short-day photoperiod significantly increased the expression of *β*-catenin from May to July and tended to increase the expression of Wnt10b from August to September, though the difference was not significant. Furthermore, in MLT implantation experiments, researchers found injection in May and June could upregulate the expression of Wnt10b and *β*-catenin (7). In present study, MLT influenced hair follicle development likely by activating the Wnt pathway. In the early stages, low levels of MLT secretion did not affect *β*-catenin gene expression, so there was no change in follicle depth and bulb width. As the experiment progressed, MLT secretion levels increased, the expression of the *β*-catenin in the SPP increased, resulting in deeper hair follicles.

PDGFA is an important gene for stimulating hair follicle development. Ponten’s research showed that PDGFA gene-knockout mice could not normally develop mature hair follicles after birth and die quickly (47). Recent studies had shown that photoperiod changes regulated the expression of PDGFA. Liu’s research (48) indicated that MLT injection had a decisive influence on the expression of key gene PDGFA in cashmere growth. Additionally, Wang (12) found that short photoperiod increased MLT concentration, which in turn upregulated PDGFA expression in hair follicles, promoting cashmere growth during the non-growth cashmere period on Shanbei cashmere goats. This was similar to the results of present study: in the end, SPP treatments increased MLT secretion, leading to an up-regulated in PDGFA expression.

BMP2 and BMP4 are members of the BMP family. Research indicated that under natural conditions, BMP2 expression varied with changes in light exposure and MLT secretion levels. As photoperiod shortened, the increasing MLT secretion lead to up-regulated BMP2 expression, during the period of hair follicle development. However, with the illumination time continued to decrease, BMP2 expression gradually declined, reaching the bottom in January, which was the resting period of hair follicle development (49). Therefore, shorten photoperiod upregulates BMP2 gene expression, showing a high-low-high pattern throughout the hair follicle development period. This was consistent with our findings, where the SPP group upregulated BMP2 expression in the later stages as MLT concentration increased, while SDPP treatment had no effect on BMP2 expression. BMP4 gene expression showed no significant changes throughout the period.

### 4.4 Photoperiod variation affect antioxidant status of skin

Currently, research on the impact of light exposure on the antioxidant status of skin was limited, with most studies combining with MLT injection. Yang (50) combined short photoperiod with MLT injection, to investigate the antioxidant capacity of skin tissue. The results showed that the treatment enhanced the antioxidant capacity of the skin tissue. This probably due to MLT directly scavenged free radicals and enhanced the activity of antioxidant enzymes. This was consistent with the results of our experiment. In present study, changes in MLT secretion by different photoperiod treatments altered the activity and gene expression of antioxidant enzymes in skin tissue. Such as, SPP increased the activity of T-SOD, CAT, and GPx, and upregulated the expression of SOD1, GPx4, and CAT genes. Similarly, previous *in vitro* experiments showed that MLT enhanced the antioxidant capacity of skin cells, specifically by increasing the activity of antioxidant enzymes and reducing the content of the MDA (51). This was also reflected in our test: the content of MDA in the skin tissue in the SDPP and SPP groups decreased at 60 d. MLT metabolites, such as 2-hydroxymelatonin, 4-hydroxymelatonin, and N1-acetyl-N2-formyl-5-methoxykynuramine (AFMK), also had strong antioxidant capacities, especially AFMK, which enhanced the antioxidant capacity of skin tissue by binding to corresponding receptors in skin cells (52). Additionally, MLT altered the balance between apoptosis and proliferation in skin cells. Studies had shown that MLT had anti-apoptotic properties in skin hair follicle mesenchymal stem cells, specifically by increasing the expression of the anti-apoptotic protein Bcl-2 and decreasing the expression of the pro-apoptotic protein Bax and Caspase-3 (53). After oxidative stress, the body induces apoptosis through the mitochondrial pathway, but antioxidant response elements (ARE) can alleviate this phenomenon (54). In present study, genes like SOD1, which were downstream of ARE, were upregulated after the photoperiod was shortened, indicating an enhancement in antioxidant capacity, which likely inhibited the apoptosis of hair follicle cells. Furthermore, Wang (55) indicated that MLT could dose-dependently reduce H2O2-induced apoptosis of skin hair follicle mesenchymal stem cells, inhibit intracellular ROS production, and increase the Bax/Bcl-2 ratio. This might indirectly explain why the depth and bulb width of secondary follicles increased at day 60. In previous studies, Yang (50) found that the beneficial effects of MLT on secondary follicles were maintained throughout life, specifically by increasing the number of secondary follicles through improved antioxidant status and inhibition of apoptosis. Similarly, MLT mediated lncRNA to elongate the cashmere fibers of secondary follicles and activated the NF-κB pathway to promote fibroblast proliferation, ultimately affecting the development of secondary follicles and cashmere growth (56).

In summary, current research indicated that hair follicle activity in cashmere goats was significantly regulated by photoperiod, with changes in illumination duration directly influencing hormone secretion, follicle morphology, gene expression, and the antioxidant state of the skin. A pathway involving MLT might explain the results of present experiment. On one hand, high concentrations of MLT reduced the serum PRL levels, increased the concentration of T3 and EGF. In addition, MLT also affected the expression of genes involved in regulating hair follicle activity. On the other hand, high concentrations of MLT enhanced the antioxidant capacity of skin tissue. This might involve upregulation of downstream molecules in the Nrf2/ARE pathway, such as the expression of the SOD1 gene, while suppressing the expression of apoptosis marker genes. Based on analysis above, the effects of photoperiod on hair follicle activity were comprehensive. For one thing, MLT regulated other hormones mediated the expression of related genes so that the hair follicle activity was influenced. For another, high concentrations of MLT enhanced the antioxidant capacity of skin tissue, which creating favorable conditions for hair follicle development.

## 5 Conclusion

This study demonstrated that shortening the photoperiod affects the secretion of hormone indicators related to hair follicle development; it could also deepen the depth and widen the bulb width of secondary follicles, and altered the expression of genes related to hair development. Additionally, shortened photoperiod improved the antioxidant status of the skin tissue of cashmere goats, creating favorable conditions for cashmere growth.

## Data Availability

The original contributions presented in the study are included in the article/supplementary material; further inquiries can be directed to the corresponding author(s).
